# Prognostic Value of Serum Cholinesterase Activity in Severe SARS-CoV-2–Infected Patients Requiring Intensive Care Unit Admission

**DOI:** 10.4269/ajtmh.21-0934

**Published:** 2022-07-25

**Authors:** Mabrouk Bahloul, Sana Kharrat, Saba Makni, Najeh Baccouche, Rania Ammar, Aida Eleuch, Lamia Berrajah, Amel Chtourou, Olfa Turki, Chokri Ben Hamida, Hedi Chelly, Kamilia Chtara, Fatma Ayedi, Mounir Bouaziz

**Affiliations:** ^1^ Department of Intensive Care, Habib Bourguiba University Hospital, and University of Sfax, Sfax, Tunisia;; ^2^ Biochemistry Laboratory, Habib Bourguiba Hospital, and University of Sfax, Sfax, Tunisia;; ^3^ Laboratory of Microbiology, Habib Bourguiba University Hospital, Faculty of Medicine, University of Sfax, Sfax Tunisia

## Abstract

We evaluated the prognostic value of serum cholinesterase (SChE) levels in SARS-CoV-2–infected patients requiring intensive care unit (ICU) admission. This is a retrospective study of severe, critically ill, adult COVID-19 patients, all of whom had a confirmed SARS-CoV-2 infection and were admitted into the ICU of a university hospital. We included all patients admitted to our ICU and whose SChE levels were explored on ICU admission and during ICU stay. One hundred and thirty-seven patients were included. There were 100 male and 37 female patients. The mean of SChE activity on ICU admission was 5,656 ± 1,818 UI/L (range: 1926–11,192 IU/L). The SChE activity on ICU admission was significantly lower in nonsurvivors (*P* < 0.001). A significant association between the SChE activity on ICU admission and the need for invasive mechanical ventilation was found. We also found a significant correlation between the SChE activity and other biomarkers of sepsis (C-reactive protein, procalcitonin, and leukocytes) on ICU admission and during the ICU stay. A significant correlation among SChE nadir value activity recorded during ICU stay, the occurrence of nosocomial infection, and the outcome of studied patients was found. Our study shows that the low SChE activity value is associated with a severe outcome. It might be used as a biomarker to aid in prognostic risk stratification in SARS-CoV-2–infected patients. Further studies for external validation of our findings are needed on this subject.

## INTRODUCTION

The newly identified coronavirus disease COVID-19 spread quickly across China and other nearby countries. To date, more than 221 countries have been affected by this pandemic, with more than 519 million infected patients reported, leading to more than 6.2 million deaths worldwide.^1^

Although fever and cough are known to be the most prevalent symptoms, severe cases of COVID-19 infection are characterized by acute respiratory failure requiring early oxygen therapy and intensive care (ICU) admission. In fact, up to 5% of SARS-CoV-2–infected patients develop severe acute hypoxemic respiratory failure requiring ICU admission as well as noninvasive and/or invasive mechanical ventilation as supportive treatment.^2^ In the ICU, biomarkers are used as adjunctive tools in clinical decision-making in various infectious diseases.^3^ Indeed, many studies have shown that elevated levels of C-reactive protein, leukocytes, and procalcitonin (PCT) in patients admitted with COVID-19 were significantly associated with higher risks of nosocomial infection and death.^3^^–^^6^ As a consequence, it is now accepted that these biomarkers might aid in prognostic risk stratification of patients with COVID-19 infection. On the other hand, serum cholinesterase (SChE) plays an important role in the inflammatory response and may be associated with prognosis in sepsis.^7^ In fact, it is well established that decreased SChE activity can be used as a reliable diagnostic biomarker for septic shock^7^ and that SChE activity level can be used as a prognostic biomarker in septic shock.^8^^,^^9^ However, there are few data published about this biomarker in patients suffering from a severe COVID-19 infection requiring ICU admission. In fact, and to the best of our knowledge, with the exception of only one study suggesting that a patient’s SChE activity level on admission may be a useful predictor of severity and prognosis,^10^ there are no other published data on this biomarker with this specific condition. In the current study, we aimed to evaluate the prognostic value of the SChE activity in SARS-CoV-2–infected patients with respiratory failure requiring ICU admission. The primary endpoint was the association between the value of the lowest value SChE and the mortality. The secondary endpoint was the association between the value of SChE and severity of illness, the development of nosocomial infection, and the correlation between the SChE activity and other biomarkers of sepsis on ICU admission and during ICU stay.

## METHODS

### Study design and setting.

This study was approved by the internal institutional review board (CPP SUD No. 0384/2022), and the requirement for written informed consent was waived by the ethics committee. It was a retrospective study of severe, critically ill adult COVID-19 patients, all of whom had a confirmed SARS-CoV-2 infection, admitted into the ICU of a university hospital between September 1 (the first case) and January 15, 2021, for acute respiratory failure. In the current study, a power calculation was not performed; as a consequence, we used the number of patients available for this analysis.

The positive diagnosis of SARS-CoV-2 infection was confirmed by reverse transcription polymerase chain reaction (RT-PCR) test in all cases. We included all patients who were admitted to our ICU and whose levels of SChE activity were explored on ICU admission and during ICU stay. Other inflammatory biomarkers (CRP, PCT, and leukocytes) were also analyzed. SChE level was measured in fresh serum that had not been frozen and thawed using a biochemistry analyzer (Cobas 6000 Analyzer, model c501) with the spectrophotometric method. The normal range of the values varies from 5,320 to 12,290 IU/L. PCT was measured with the electrochemiluminescence method using a Cobas 6000 Analyzer (model e601), and CRP was measured by using the immunoturbidimetric method. All biomarker assays were processed at the same laboratory.

SChE activity was recorded on ICU admission for all patients and during the ICU stay if it was performed. CRP and PCT were also recorded on admission and during ICU stay, if performed. In our ICU, all inflammatory biomarkers were explored on ICU admission, 72 hours after ICU admission (if possible), and as needed (if the patient developed a fever or when a nosocomial infection was suspected). For each patient, the nadir of inflammatory biomarkers was defined as the highest value during ICU stay for CRP, leukocytes, and PCT. The nadir of SChE activity was defined as the lowest recorded value during ICU stay.

Data collected for all hospitalized COVID-19 patients included clinical, radiological, and treatment data on the day of admission and during ICU stay. Body mass index^11^ (BMI) was measured on admission based on height and weight that apply to adult men and women. Obesity^11^ was defined as BMI > 30 kg/m^2^.

Data collection also included laboratory findings on ICU admission and during ICU stay. Treatment modalities, complications during hospitalization, and discharge status were also recorded. The severity of illness was evaluated by the Sepsis-related Organ Failure Assessment (SOFA) score calculated on ICU admission and during ICU hospitalization,^12^ and according to the Simplified Acute Physiology Score (SAPS II) assessed within 24 hours of admission.^13^ During the ICU stay, all complications were recorded: nosocomial infections (hospital-acquired/associated infections),^14^ gastrointestinal bleeding, barotrauma, arrhythmias, renal failure, and metabolic complications. Acute respiratory distress syndrome (ARDS) was defined according to the recently published formal guidelines.^15^ According to the PaO_2_/FiO_2_ ratio, ARDS was classified as mild (200 < PaO_2_/FiO_2_ ≤ 300 mm Hg), moderate (100 < PaO_2_/FiO_2_ ≤ 200 mm Hg), or severe (PaO_2_/FiO_2_ ≤ 100 mm Hg). Diagnosis of shock^16^ was based on clinical, hemodynamic (systolic arterial pressure < 90 mm Hg or mean arterial pressure < 70 mm Hg, with associated tachycardia), and biochemical signs.

All parameters were recorded from ICU admission until ICU discharge or death.

### Statistical modeling.

All statistical analyses were performed using the IBM SPSS Statistics for Windows, version 20 (IBM Corp., Armonk, NY). Categorical data were expressed in proportion and subgroups, and continuous variables were expressed as means (±SD) and/or median. Subgroups were analyzed by the chi-square test (or Fisher’s exact test), the two-group *t*-test, or the Mann–Whitney *U* test, as appropriate. To analyze the relationship between the SChE activity on ICU admission or the nadir SChE activity and other continuous variables, we used Pearson’s correlation coefficient (*r*). For all used tests, the level of significance was set at *P* < 0.05. Receiver operating characteristic (ROC) curves were plotted, and the respective areas under curves were calculated. The SChE nadir was used to predict poor outcome (death and nosocomial infections) and analyzed using ROC curves. The area under the ROC curve (AUC), which was estimated by the method of Hanley and McNeill,^17^ provides a measure of the predictive accuracy of the test. The best cutoff values for serum SChE activity was chosen as the values that optimized sensitivity and specificity: the value that maximizes (sensitivity + specificity – 1).

## RESULTS

### Clinical and biological findings of the entire population group.

During the study period, 178 patients were admitted into our ICU for acute respiratory distress due to SARS-CoV-2 infection. Forty-one patients were excluded because SChE level was not recorded on ICU admission. As a result, only 137 patients were included. There were 100 male and 37 female patients. Mean age was 62.6 ± 12.3 with a median of 65 years. Fifty-three patients (38.6%) were obese with a BMI > 30 kg/m^2^, and 111 patients (81%) had one or more comorbidities. The most commonly observed comorbidities were arterial hypertension in 65 patients (47.4%), diabetes mellitus in 62 (45.3%), and chronic obstructive pulmonary disorder in 13 (9.5%). The most common symptoms observed on hospital admission were dyspnea in 127 patients (92.7%), fever (≥ 38°C) in 88 (64.2%), and cough in 84 (61.8%). The mean time from the onset of symptoms to ICU admission was at 10.9 ± 5.5 days.

On ICU admission, all the patients had major signs of respiratory distress. The mean respiratory rate was at 30 ± 11 breaths per minute; the mean oxygen saturation measured by pulse oximetry (SpO_2_) was at 87 ± 11% (under oxygen support via facial mask). The mean body temperature was at 37 ± 0.8°C; fever (≥ 38°C) was observed in only 20 patients (14.5%). Moreover, 16 patients (11.6%) developed shock and the use of vasopressor (norepinephrine) was required in all these cases. The mean PaO_2_/FiO_2_ ratio was at 100 ± 58 with a median at 82. It was < 200 in 128 patients (93.4%). On the day of ICU admission, oxygen therapy was required for all patients (100%), via facial mask for 109 (79.6%), noninvasive mechanical ventilation for 82 (60%), and high-flow nasal oxygen for 57 (41.6%). Invasive mechanical ventilation was applied in 28 patients on the day of ICU admission and in 64 (46.7) during ICU stay. The mean pH on ICU admission was at 7.39 ± 0.10, ranging from 6.90 to 7.58. Among the inflammatory parameters recorded on ICU admission for patients with available data, the mean value of leukocytes was at 13,650 ± 5,960 cells/mm^3^. Mean CRP was at 120 ± 106 mg/L, it was ≥ 100 mg/L in 42% of the cases. The mean procalcitonin was at 2.46 ± 10 ng/mL, ranging from 0.1 to 100 ng/mL. Finally, the mean of SChE activity was at 5,656 ± 1,818 IU/L (range: 1,926–11,192 IU/L). Table 1 summarizes all the clinical and biological findings of the entire population group on ICU admission. During their ICU stay, 63 patients (46%) developed shock requiring the use of vasopressor (norepinephrine), 78 developed renal failure (57%), 64 (46.7%) required the use of invasive mechanical ventilation, and 63 (46%) developed nosocomial infections. Corticosteroids (dexamethasone, 12–24 mg/day) were given to 130 patients (95%), and preventive and/or therapeutic anticoagulation for venous thromboembolism were used in 136 patients (99%). However, remdesivir/tocilizumab was not used during the study period.

**Table 1:** Clinical and laboratory findings of the study population at the time of intensive care unit admission

Parameters	Data available for (n)	Mean ± SD/proportion (%)	Normal ranges
Age (years)	137	62 ± 12	–
Obesity (body mass index > 30)	137	54 (39.4)	–
Diabetes mellitus	137	62 (45.3)	–
Arterial hypertension	137	65 (47.4)	–
Chronic heart disease	137	49 (35.8)	–
Chronic obstructive pulmonary disease	137	13 (9.5)	–
SAPS-II	137	34.2 ± 14.4	–
SOFA score	137	4.9 ± 2.6	–
Blood glucose level (mmol/L)	137	12.6 ± 6.2	4.0–6.1
White blood cell count (cells/mm^3^)	133	13,650 ± 5,960	4,000–10,000
Lymphocytes (cells/mm^3^)	100	816 ± 851	1,500–4,500
Hemoglobin (g/dL)	132	12.6 ± 1.9	13.0–16.7
Platelets count (cells/mm^3^)	132	309,977 ± 126,789	150–439 × 10^3^
ASAT (IU/L)	131	44 ± 37	10–37
ALAT (IU/L)	128	40 ± 54	10–41
Total bilirubin (μmol/l)	123	10 ± 8	< 25
Lactate (mmol/l)	7	2.3 ± 0.7	< 2
Total protein (g/l)	119	69 ± 7.9	60–80
D-dimer (ng/ml)	19	1,545 ± 1,901	< 500
Prothrombin ratio (%)	116	77 ± 16.5	70–100
Troponin (ng/ml)	126	0.04 ± 0.08	< 0.014
Pro-BNP (pg/ml)	110	2,065 ± 5,307	< 150
pH	133	7.39 ± 0.1	7.37–7.43
PaCO_2_ (mm Hg)	133	38 ± 10	37–43
PaO_2_ (mm Hg)	133	73 ± 26	80–100
HCO_3_- (mmol/L)	132	23 ± 5	24–26
PaO_2_/FiO_2_ ratio	133	100 ± 58	400–500
Urea (mmol/L)	137	13.7 ± 11.8	2.5–8.0
Creatinine (μmol/L)	137	124.5 ± 150	62–106
Procalcitonin (µg/L) on ICU admission	112	2.46 ± 10.6	< 0.01
C-reactive protein (mg/L) on ICU admission	129	120 ± 106	< 6
SChE activity (IU/L) on ICU admission	137	5,656 ± 2,279	5,320–12,290
Highest procalcitonin value (?g/L) during ICU stay	130	3.4 ± 10.6	< 0.01
Highest CRP value (mg/L) during ICU stay	131	192 ± 150	< 6
Lowest SChE activity value (IU/L) during ICU stay	137	4,950 ± 1,837	5,320–12,290

ALT = alanine transaminase; AST = aspartate aminotransferase; FiO_2_ = fractional inspired oxygen; HCO3– = bicarbonate; Pro-BNP: pro-brain natriuretic peptide; PaCO_2_ = arterial carbon dioxide tension; PaO_2_: arterial oxygen tension; SAPS = Simplified Acute Physiology Score; SChE = serum cholinesterase; SOFA = Sequential Organ Failure Assessment.

The most observed nosocomial infections were ventilator-associated pneumonia in 38 patients (28%), urinary tract infection in 12 (9%), and septicemia in 7 (5.1%). During their ICU stay, 74 patients (54%) died, with an average time from ICU admission to death at 9.7 ± 6.4 days (median: 8 days). Thirty-four patients (25%) died within 7 days after ICU admission, of whom 21 had developed nosocomial infections. On the other hand, 40 patients (29.2%) died after 7 days of ICU admission, of whom 27 had developed nosocomial infections.

The mean duration of ICU stay was at 8.4 ± 5.9 days. Supplemental Table 1 shows the comparison between patients according to the severity of the ARDS. Supplemental Table 2 shows the comparison between the Survivors and Deaths groups according to the severity of the ARDS.

### Prognostic value of the SChE activity on ICU admission.

The comparison between two groups (Survivors and Deaths) on ICU admission showed that the SChE activity is significantly lower in nonsurvivors (6,273 ± 1,823 versus 5,138 ± 1,655 IU/L; *P* < 0.001). A value of SChE activity < 6,000 IU/L was associated with a severe outcome with sensitivity at 58%, specificity at 76%, and an AUC at 0.70.

We found a significant association between the SChE activity on ICU admission and the need for invasive mechanical ventilation (6,238 ± 1,885 versus 5,039 ± 1,520; *P* < 0.001). We found that the value of the SChE activity on ICU admission was correlated with the SOFA score (*P* < 0.001; *r* = –0.3) and the SAPS II score (*P* < 0.001; *r* = –0.38). Finally, we found a significant correlation between the SChE activity and other biomarkers of sepsis on ICU admission—in particular, with CRP (*P* = 0.02; *r* = –0.2), leukocytes (*P* = 0.002; *r* = –0.27), and procalcitonin (*P* = 0.03; *r* = –0.20).

### Prognostic value of the nadir of the SChE activity.

**Figure 1.**
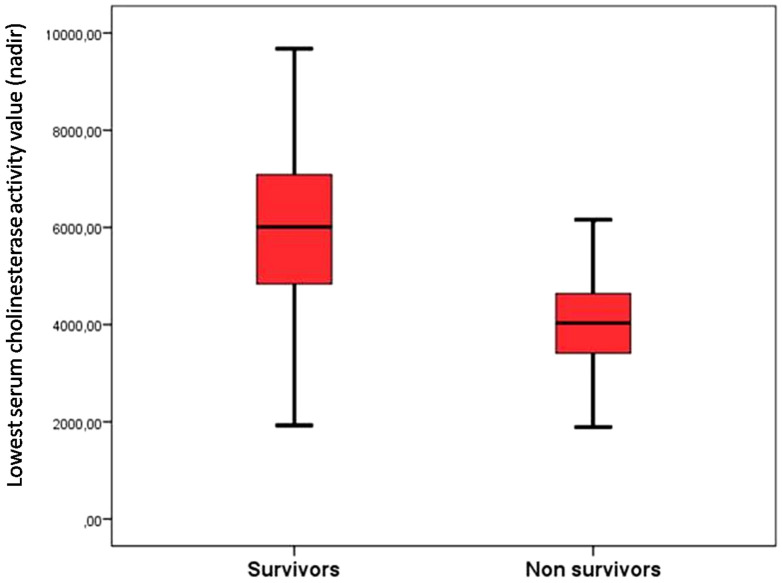
Lowest serum cholinesterase activity (nadir) stratified by outcome. This figure appears in color at www.ajtmh.org.

**Figure 2.**
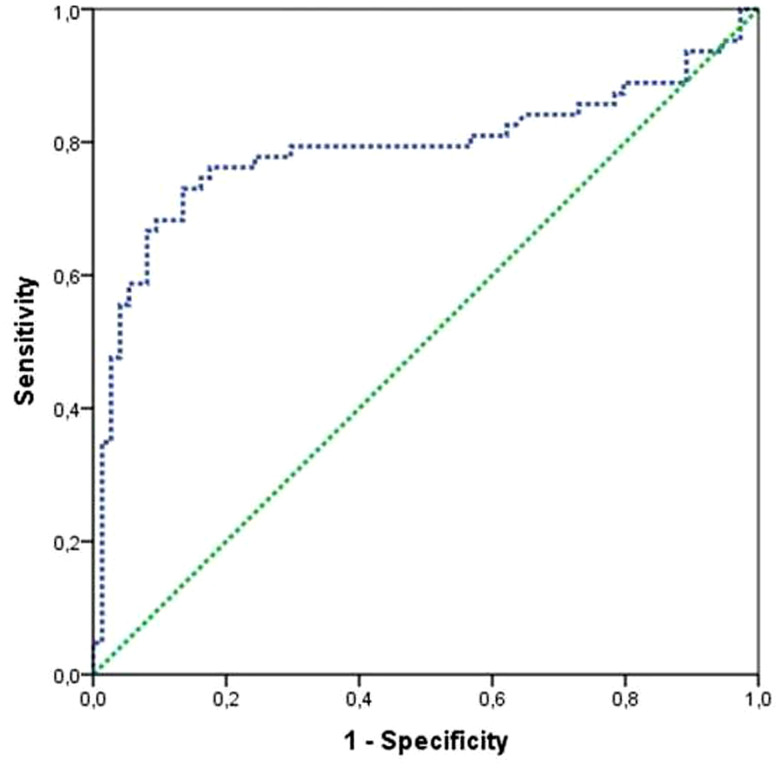
Receiver-operating characteristic curve for ability of association of serum cholinesterase activity with mortality. The area under the curve was 0.80, indicating a good capability of the model to discriminate between survivors and nonsurvivors. This figure appears in color at www.ajtmh.org.

The comparison between two groups (Survivors and Deaths) showed that the SChE activity is significantly lower in nonsurvivors (5,960 ± 2,008 versus 4,091 ± 1,111 IU/L; *P* < 0.001) (Figure 1). A value of SChE activity < 5,000 IU/L was associated with a severe outcome with sensitivity at 71%, specificity at 87%, and an AUC at 0.80 (95% confidence interval [CI]: 0.70–0.87) (Figure 2). Multivariate analysis showed that factors associated with poor outcomes were: age > 65 years (*P* = 0.016; odds ratio [OR]: 8.3; 95% CI: 1.49–46.4), the use of mechanical ventilation (*P* < 0.001; OR: 37; 95% CI: 5.89–134.3), and a value of SChE activity lower than 5,000 IU/L (*P* = 0.037; OR: 6.3; 95% CI: 1.12–35.90).

**Figure 3.**
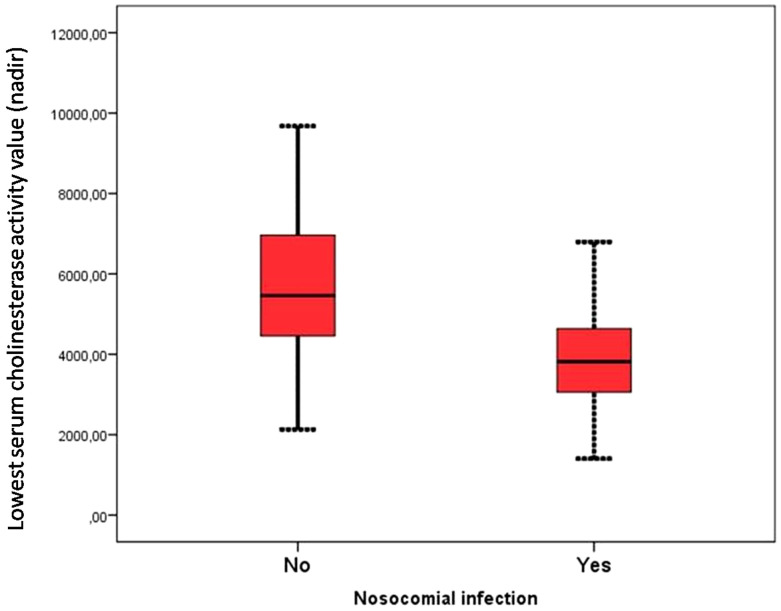
Lowest serum cholinesterase activity (nadir) stratified by the development of the nosocomial infection. This figure appears in color at www.ajtmh.org.

We found that the value of the SChE activity was significantly lower in the group of patients who developed septic shock requiring the use of vasopressor (5,711 ± 1,975 versus 4,057 ± 1,144 IU/L; *P* < 0.001) and nosocomial infection (5,772 ± 1,869 versus 3,986 ± 1,241 UI/L; *P* < 0.001) (Figure 3). The value of SChE activity was significantly correlated with the severity of hypoxemia and the PaO2/FiO2 ratio (*P* = 0.003; *r* = 0.25).

Finally, we found a significant correlation between the nadir of SChE activity (lowest value) recorded during ICU stay and the nadir recorded for other biomarkers of sepsis—in particular, with CRP (*P* < 0.001; *r* = –0.38) and procalcitonin (*P* = 0.04; *r* = –0.17).

## DISCUSSION

Our study showed that the SChE activity might be used as a new biomarker that helps in the prognostic risk stratification of patients with COVID-19 infection. In fact, we found that a low value of SChE activity recorded on ICU admission and during ICU stay was associated with a poor prognosis in univariate (in all studied population and in each subtype of ARDS: mild, moderate, and severe) and multivariate analyses. Moreover, we found a significant association between the SChE activity on ICU admission and the severity of illness (SAPS II and SOFA score). We found again a significant correlation between the value of SChE activity and the development of nosocomial infection. Finally, we found a significant correlation between the SChE activity and other biomarkers of sepsis on ICU admission and during ICU stay. These significant correlations confirm that this biomarker could help prediction, together with other markers (biomarkers and digital markers such as SAPS, SOFA, and ARDS severity), to predict outcome. In fact, in our study, an SChE activity < 5,000 IU/L was associated with a poor outcome with high sensitivity and specificity. The cost of this biomarker is low (< $5), and it is usually used in the diagnosis and management of organophosphate poisoning,^18^ particularly in low- and middle-income countries.

It has been established that many inflammatory (procalcitonin, CRP) and biochemical (troponin I, lactate dehydrogenase, D-dimer, gamma-glutamyl transferase) biomarkers are significantly associated with a severe form of COVID-19 infection.^3^^–^^6^^,^^19^^,^^20^ Moreover, it has already been established that elevated levels of CRP, leukocytes, and PCT in patients admitted with COVID-19 are significantly associated with a higher risk of nosocomial infection and death in SARS-CoV-2–infected patients.^3^^–^^6^ However, the correlation between the cholinesterase activity and the severity of COVID-19 pneumonia is not often reported. In fact, and to the best of our knowledge, only one study^10^ investigated this association exclusively. Indeed, Nakajima et al.^10^ found in their study that cholinesterase levels on admission were significantly associated (326 versus 218 IU/L, *p* = 0.006) with the severity of COVID-19 pneumonia in 26 patients with confirmed COVID-19 infection. They found again that cholinesterase levels on admission were significantly lower in the Deaths group than in the Survivors group.^10^ The authors concluded that the SChE activity may reflect the disease state of COVID-19 pneumonia and suggest that the cholinesterase level on admission may be used as one of the predictors of severity and prognosis. In one retrospective study analyzing the clinical, radiological, and biological characteristics, and the outcome of critically ill patients admitted to the ICU for acute respiratory failure following COVID-19 infection,^21^ we found that SChE activity lower than 5,000 IU/L was associated with a severe outcome. However, this study^21^ included only 96 patients, among whom only 78 patients had an SChE activity recorded on ICU admission. In the current study, a total of 178 patients were admitted into our ICU for respiratory distress due to COVID-19 infection. However, only 137 patients were included because 41 patients were excluded because the level of the SChE activity was not recorded on ICU admission. The impact outcome of the lowest value of SChE activity is more extensively studied in the current study. In fact, we have analyzed the association between the SChE activity on ICU admission and the severity of illness (SAPS II and SOFA score). In the current study, we found that the SChE activity may reflect the disease state of COVID-19 pneumonia; therefore, we suggest that the cholinesterase level on admission and during ICU stay may be used as one of the predictors of severity and prognosis. We also found a significant correlation between the value of SChE activity and the development of nosocomial infection, the development of shock, and the other biomarkers of sepsis on ICU admission and during ICU stay.

Recently, it was established that a decreased SChE activity can be used as a diagnostic biomarker for septic shock and bacterial infection.^2^^,^^8^ The pathophysiology of the decline of the SChE activity in septic patients is not well understood, and several hypotheses have been postulated.^7^ In all cases, the role of inflammatory reaction is well established.^7^^–^^10^ The first hypothesis is reduced synthesis of SChE due to bacterial and/or viral infection. The second is the increase of SChE catabolism and the inhibition of SChE by inflammatory mediators (cytokines). In patients suffering from severe COVID-19 pneumonia requiring ICU admission, the infection of alveolar units by the SARS Cov-2 virus leads to diffuse alveolar damage with an important inflammatory response and a secondary cytokine (tumor necrosis factor-α and interleukin-6) storm^22^ leading to inhibition of SChE. Another mechanism should be considered in severely SARS-CoV-2 infected patients: the severity of hypoxemia leading to liver injury and affliction. We found a significant correlation between the value of the SChE activity and the severity of hypoxemia and the PaO_2_/FiO_2_ ratio (*P* = 0.003; *r* = 0.25). In our study, although most patients received corticosteroids and anticoagulation (95% and 99%, respectively), the majority exhibited a severe inflammatory response triggered by SARS-COV-2. This confirms the severity of this inflammatory response; in fact, like corticosteroids, anticoagulants were found to have an interesting antiinflammatory action.^23^

Finally, we acknowledge that our study suffers has limitations. All retrospective studies suffer from incomplete information. CRP and PCT tests were not performed for all patients at ICU admission. Moreover, the time that SChE activity nadir was determined was not the same for all patients. The number of patients that could not be included was high, which can cause bias in interpretation of the results.

However, our study is one of the few studies that has dealt with the prognostic impact of the SChE activity in patients suffering from severe COVID-19 infection with acute respiratory failure. Consequently, we think that SChE activity can be used in the screening of severe patients in ICU and in the emergency department because it is correlated with routinely monitored inflammatory parameters. Moreover, the reduction in the SChE activity appears significantly earlier (within 1–2 hours after onset of inflammation or sepsis) than those of routinely measured inflammatory biomarkers.^24^ Finally, we recommend routine monitoring of SChE activity in the ICU because the SChE activity level has been used as a prognostic biomarker in other studies, particularly for septic shock.^7^^–^^9^

## CONCLUSION

Our study shows that the low SChE activity is associated with a poor outcome, development of nosocomial infections, and illness severity. It might be used as a new biomarker to help in the prognostic risk stratification of patients with COVID-19 infection. Further studies for external validation of our findings are needed on this subject.

## Supplemental files


Supplemental materials
