# Supplementary material for: Prognostic Value of Serum Cholinesterase Activity in Severe SARS-CoV-2–Infected Patients Requiring Intensive Care Unit Admission
Source: Am J Trop Med Hyg. 2022 Jul 25;107(3):534–9. doi: 10.4269/ajtmh.21-0934 (PMC9490658; doi:10.4269/ajtmh.21-0934)
Supplement: Supplementary file 1 [file tpmd210934.SD1.pdf]

Supplemental Table S2. Comparaison of patients according the severity of the acute respiratory distress syndrome

Parameters	Midl ARDS (G1 ; N=14)	Moderate ARDS (G2 ; N=23)	Severe ARDS (G3; N=100)	P G1 vs G2	P G1 vs G3	P G2vs G3
Age (years)	63 ± 13	61 ± 11	63 ± 12	0.58	0.91	0.52
Obesity (BMI>30)	4(28.5%)	11(47.8%)	39(39%)	0.24	0.45	0.43
Diabetes mellitus	5(35.7%)	11(47.8%)	46(46%)	0.47	0.45	0.87
Arterial hypertension	3(21.4%)	10(43.4%)	52(52%)	0.17	0.03	0.46
Chronic heart disease	2(14.2%)	9(39.1%)	39(39%)	0.1	0.07	0.99
COPD	3(21.4%)	1(4.3%)	9(9%)	0.1	0.15	0.46
GCS	12± 4	14 ± 3	14 ± 3	0.051	0.15	0.59
SpO2 under O2	92 ± 7	87 ± 15	86 ± 10	0.29	0.06	0.76
Body temperature (°C)	37.2 ± 1	37.1 ± 0.6	37 ± 0.8	0.52	0.31	0.71
SAPSII Score	34 ± 20	32 ± 14	34 ± 13	0.82	0.83	0.51
SOFA Score	5 ± 4	4 ± 2	5 ± 2	0.14	0.7	0.04
PCT (µg/L) on ICU admission	6.7±13.6	0.7 ± 1.6	2.2 ± 11.2	0.06	0.19	0.57
CRP (mg/L) on ICU admission	121 ± 123	106 ± 82.5	123 ± 109	0.65	0.95	0.48
SChE activity (UI/L) on ICU admission	6108 ± 2153	5739 ± 2100	5573 ± 1706	0.61	0.29	0.68
Highest PCT value (µg/L)	7.6 ± 13.3	2.1 ± 6.2	3.1 ± 10.9	0.12	0.17	0.70
Highest CRP (mg/L)	145 ± 126	156 ± 105	207 ± 159	0.79	0.20	0.15
lowest SChE activity value (UI/L)	5668 ± 2192	5365 ± 2004	4727 ± 1704	0.48	0.02	0.12
Deaths/Survivors	7/7	5/18	62/38	0.07	0.39	0.00
Invasive MV (Yes/No)	5/9	6/17	53/46	0.53	0.21	0.18
PCT (µg/L) on ICU admission in deaths	5.8±9	1.4±2.8	3.3±14.1	0.32	0.65	0.77
CRP (mg/L) on ICU admission in deaths	164±153	164±115	146±120	0.99	0.72	0.74
SChE activity (UI/L) on ICU admission in deaths	4920±2252	5669±2279	5120±1553	0.58	0.75	0.46
Highest PCT value (µg/L) in Deaths	7.4±8.3	6.7±11.5	4.5±13.5	0.91	0.58	0.71
Highest CRP value (mg/L) in deaths	181±152	225±94	250±169	0.36	0.31	0.95
lowest SChE activity value (UI/L) in deaths	4439±1991	4213±949	4042±1005	082	0.38	0.71
ICU Stay (days)	6.7 ± 6.8	6.6 ± 4.5	9 ± 6	0.95	0.19	0.07

G1 : Group1, G2 : Group2, G3 : Group3, COPD: Chronic obstructive pulmonary disease; SAPS: Simplified Acute Physiology Score; SOFA: Sequential Organ Failure Assessment;   GCS: Glascow Coma Scale;   PCT : Procalcitonin ; CRP : C-reactive protein; MV: mechanical ventilation

Supplemental Table S3. Comparison of characteristics of survivors and deceased patients according to the severity of acute respiratory distress syndrome

	Parameters	Survivors	Non survivors	p
All Population group	SAPSII Score	29 ±11	38 ±15	<0.001
	SOFA Score	4 ± 2	5 ±3	<0.001
	PCT (µg/L) on ICU admission	1.31±6.53	3.4±13.05	0.3
	CRP (mg/L) on ICU admission	86.36±71.51	149.67±122.2	0.01
	SChE activity (UI/L) on ICU admission	6273±1823	5138±1655	<0.001
	WBC count (cells/mm^3) on ICU admission	12992±5262	14190±6464	0.25
	Highest PCT value (µg/L)	1.43±6.22	3.4±12.95	0.06
	Highest CRP value(mg/L)	128.15±99.94	244.32±163.81	<0.001
	Lowest SChE activity value(UI/L)	5960±2008	4091±1111	<0.001
Group 1 (mild ARDS)	SAPSII Score	19 ±8	48 ±19	0.001
	SOFA Score	2±1	8 ±4	0.001
	PCT (µg/L) on ICU admission	7.96±18.64	5.76±8.96	0.78
	CRP (mg/L) on ICU admission	72.13±55.01	164.14±153.04	0.19
	SChE activity (UI/L) on ICU admission	7296±1297	4920±2252	0.03
	WBC count (cells/mm^3) on ICU admission	9131±6773	13971±10068	0.31
	Highest PCT value (µg/L)	7.97±18.64	7.43±8.3	0.94
	Highest CRP value(mg/L)	94.6±59.27	181.85±152.5	0.25
	Lowest SChE activity value(UI/L)	7296±1297	4439±1991	0.00
Group 2 (moderate ARDS)	SAPSII Score	30±12	41±19	0.14
	SOFA Score	4 ± 2	4 ± 2	0.47
	PCT (µg/L) on ICU admission	0.44±1.07	1.44±2.81	0.26
	CRP (mg/L) on ICU admission	90±66.09	164.8±115.97	0.07
	SChE activity (UI/L) on ICU admission	5758±2117	5669±2279	0.94
	WBC count (cells/mm^3) on ICU admission	13072±5072	11480±5214	0.54
	Highest PCT value (µg/L)	0.44±1.07	1.44±2.81	0.04
	Highest CRP value(mg/L)	126.8±91	255.4±94.1	0.01
	Lowest SChE activity value(UI/L)	5685±2118	4213±949	0.015
Group 3 (severe ARDS)	SAPSII Score	30 ±11	37 ±14	0.01
	SOFA Score	4 ±2	5 ±3	0.04
	PCT (µg/L) on ICU admission	0.4±1.1	3.23±14.16	0.27
	CRP (mg/L) on ICU admission	86.91±77.62	146.56±120.87	0.01
	SChE activity (UI/L) on ICU admission	6331±1701	5120±1553	0.00
	WBC count (cells/mm^3) on ICU admission	13722±4844	14438±6124	0.55
	Highest PCT value (µg/L)	0.72±1.61	4.51±13.56	0.09
	Highest CRP value(mg/L)	133.46±108.67	250.58±169.64	<0.001
	Lowest SChE activity value(UI/L)	5844±2009	4042±1005	<0.001

SAPS: Simplified Acute Physiology Score; SOFA: Sequential Organ Failure Assessment; PCT : Procalcitonin ; CRP : C-reactive protein; MV: mechanical ventilation
